# The Development and Validation of Measurement Scales of Upward State Social Comparison on Social Media

**DOI:** 10.3390/bs15060743

**Published:** 2025-05-27

**Authors:** Muheng Yu, Drew P. Cingel

**Affiliations:** 1 School of Journalism, Communication University of China, No. 1 Dingfuzhuang East Street, Chaoyang District, Beijing 100024, China; 2 Department of Communication, University of California Davis, One Shields Avenue, Davis, CA 95616, USA; dcingel@ucdavis.edu

**Keywords:** measurement, psychological well-being, social media use, upward state social comparison, validation

## Abstract

In the literature of social media use, upward state social comparison, and psychological well-being, there are multiple issues on how upward state social comparison is measured. Although researchers have pointed out these measurement issues, existing research has not provided specific measurement recommendations or measurement scales to address them. In this paper, we offered multiple specific recommendations on how to measure upward state social comparison, and we also developed measurement scales. Lastly, in a validation study, we validated the measurement scales we developed in a sample of young adult social media users in the U.S. The final sample size was 462 participants, with ages ranging from 18 to 42 years old, (*M*_age_ = 19.75, *SD*_age_ = 2.09), and the gender distribution was 349 females (75.54%), 105 males (22.73%), four participants identifying with other genders (0.87%), and four participants preferring not to reveal their gender (0.87%). Results of the validation study showed that a 4-item scale of measuring ability-based assimilative upward state social comparison and a 4-item scale of measuring ability-based contrastive upward state social comparison demonstrated good reliability, convergent validity, divergent validity, and predictive validity. Therefore, in this paper, we offered promising suggestions and tools to solve multiple measurement issues of upward state social comparison.

## 1. Introduction

Abundant studies have shown that upward state social comparison could explain how social media use negatively affects psychological well-being (for a review, see Verduyn et al., 2017, 2020). However, it should be noted that existing research has multiple issues in terms of measuring upward state social comparison, and these measurement issues could potentially confound the findings in existing research (Johnson, 2021; Meier & Johnson, 2022). Therefore, it is important to figure out how to potentially solve these measurement issues.

Existing research, although already pointing out multiple measurement issues of upward state social comparison (e.g., for a review, see Meier & Johnson, 2022), has not yet synthesized these measurement issues to offer specific recommendations or concrete measurement scales to solve them. Therefore, in this paper, we first review the literature on how upward state social comparison could explain the relationship between social media use and negative psychological well-being, and review multiple measurement issues involving upward state social comparison. Next, addressing all measurement issues reviewed, we provide multiple specific recommendations on how to measure upward state social comparison on social media, and propose concrete measurement scales. Lastly, we validate the proposed scales.

### 1.1. Upward State Social Comparison on Social Media

Social comparison theory (Festinger, 1954) proposes that individuals have a natural tendency to compare themselves with other people. Social media provide abundant opportunities for social comparison. With just a few clicks, individuals can easily see what other people are doing and how other people are doing (Verduyn et al., 2017). Therefore, on social media, when individuals see the content posted by other people, in the moment, individuals tend to compare themselves with other people. This is referred to as state social comparison on social media (e.g., Lee, 2014).

Furthermore, research has shown that state social comparison on social media tends to be upward in nature. That is, when individuals engage in social comparison at the moment when they see other people’s content, they are likely to compare themselves with other people whom they perceive to be superior (Verduyn et al., 2020). On social media, users can implement various techniques to engage in selective and strategic self-presentation to make their lives appear to be very happy and successful (known as the positivity bias on social media; Schreurs & Vandenbosch, 2021). Therefore, when individuals engage in social comparison on social media, in the moment, they are likely to compare themselves with the positively biased content of other people. As such, state social comparison on social media tends to be upward (Verduyn et al., 2017).

### 1.2. Passive Use, Upward State Social Comparison, and Psychological Well-Being

Research has shown that a particular type of social media use––passive social media use––is especially likely to prompt upward state social comparison, which in turn could negatively affect psychological well-being (for a review, see Verduyn et al., 2017, 2020). Passive social media use refers to individuals browsing and seeing the content posted by others, without any interactions with others (Frison & Eggermont, 2020). When individuals passively use social media, they are seeing other people’s content on social media. In the moment, it is natural for them to engage in upward state social comparison, and such upward state social comparison, in turn, could negatively affect psychological well-being (e.g., Schmuck et al., 2019). In summary, numerous existing studies have shown that upward state social comparison could explain why passive social media use could have negative effects on psychological well-being.

### 1.3. Measurement Issues on Upward State Social Comparison

Despite these findings, it should be noted that existing research faces multiple issues involving the measurement of upward state social comparison, potentially confounding the findings in existing research (Johnson, 2021; Meier & Johnson, 2022). In this section, we review a total of five measurement issues identified in existing research.

#### 1.3.1. Ability-Based Versus Opinion-Based Social Comparison

The first measurement issue of upward state social comparison deals with the entanglement of ability-based social comparison and opinion-based social comparison. It should be noted that there is also another type of social comparison––appearance-based social comparison. However, appearance-based social comparison is often conceptualized and measured independently and separately from ability-based and opinion-based social comparison, and appearance-based social comparison already has its own well-validated and highly reliable measurement scales (e.g., Jarman et al., 2021; O’Brien et al., 2009). Therefore, appearance-based social comparison is not entangled with ability-based and opinion-based social comparison. The focus of this paper is the entanglement issue of ability-based and opinion-based social comparison.

Ability-based social comparison refers to people’s tendency of evaluating how well (e.g., better or worse, superior or inferior) they are doing, in comparison with others on social media (Yang & Robinson, 2018). For example, on social media, individuals may compare their own lifestyle with the lifestyle of others, in order to evaluate how well their own lives are (Choi & Kim, 2021). Ability-based social comparison on social media is judgmental in nature (i.e., individuals judge whether they are doing better or worse than others) and involves directions (i.e., upward comparison with other people who are superior, downward comparison with other people who are inferior; e.g., Gerber et al., 2018; Park & Baek, 2018). On the other hand, opinion-based social comparison refers to people’s tendency of comparing what others think on certain issues with what they themselves think to inform on their own opinions on social media (Park & Baek, 2018). For example, college students compare their own opinions with the opinions of their peers on social media to learn whether their opinions are close to or different from others’, helping them bond with their peers in college (Yang et al., 2018). Unlike ability-based social comparison, opinion-based social comparison is non-judgmental and non-directional—individuals do not judge whose opinions on a certain issue/phenomenon are better/superior or worse/inferior (e.g., Gerber et al., 2018; Yang & Robinson, 2018).

In the existing literature, a lot of researchers conceptualize upward state social comparison on social media as ability-based (e.g., for a review, see Verduyn et al., 2017), and it should be acknowledged that some researchers clearly measure upward state social comparison as ability-based in some specific social media contexts, such as adolescent peer popularity and online dating (e.g., Boer et al., 2021; Thomas et al., 2023). However, some researchers, despite conceptualizing upward state social comparison on social media as ability-based, measure it using both ability-based and opinion-based measurement items (e.g., Jang et al., 2016; Jiang & Ngien, 2020; Stapleton et al., 2017). In other words, at times, the measurement of ability-based upward state social comparison entangles both ability-based items and opinion-based items. This entanglement issue could confound the findings in existing research. That is, it is unclear whether the observed relationships between social media use and psychological well-being are indeed explained by ability-based upward state social comparison, or explained by opinion-based social comparison, or explained by both.

#### 1.3.2. Upward Directionality of Social Comparison

The second measurement issue of upward state social comparison is that sometimes researchers do not specify upward directionality in the measurement items of upward state social comparison. For example, Rousseau et al. (2017) argued that state social comparison on Facebook is upward but ended up measuring state social comparison without specifying the upward directionality, by using items such as “I often compare myself with others on Facebook when I am reading news feed or checking others’ photos” (p. 339) (see also Jang et al., 2016; Ozimek & Bierhoff, 2020). Without specifying the upward directionality in the measurement items, it is not clear whether it is indeed the upward nature of state social comparison that explains the observed relationships between social media use and psychological well-being, or it is other directions (e.g., downward) of state social comparison that explain these relationships. In this sense, the findings of existing studies could be confounded.

#### 1.3.3. State Versus Trait Social Comparison

The third measurement issue of upward state social comparison is that sometimes researchers measure upward state social comparison as a trait, not as a state (Johnson, 2021; Meier & Johnson, 2022). As reviewed previously, upward state social comparison is a state––individuals compare themselves with those superior others at the moment when they see the social media content posted by other people (e.g., Lee, 2014). Upward state social comparison is not a trait (Johnson, 2021; Meier & Johnson, 2022). That is, upward state social comparison is not an individual’s general inclinations or habits of comparing themselves with those superior others on social media.

Some researchers, when measuring upward state social comparison, use items such as “on social network sites, I always like comparing myself with others who are better off” (Liu et al., 2017, p. 225); “on social network sites, I often compare myself with those who are more successful than me” (He et al., 2020, p. 3; see also Zheng et al., 2020). These items tend to reflect a trait (on social media, I often/always (i.e., have a general habit to) compare myself with people who are superior to me), not a state. If researchers measure upward state social comparison as a trait but not a state, the observed relationships between social media use and psychological well-being might not be accurately explained by a person’s upward state social comparison, but rather could be confounded by a person’s general trait of upward social comparison.

#### 1.3.4. Assimilative and Contrastive Social Comparison

Another measurement issue on upward state social comparison is that researchers often fail to differentiate the nuances between different subtypes of upward state social comparison (Meier & Johnson, 2022). There are two different subtypes of upward state social comparison—assimilative upward state social comparison and contrastive upward state social comparison. Assimilative upward state social comparison refers to individuals comparing themselves with those superior others on social media to focus on how they can be similar to those superior others; on the other hand, contrastive upward state social comparison refers to individuals comparing themselves with those superior others on social media to focus on how they are different from those superior others (Collins, 1996; Suls et al., 2002; Tsay-Vogel & Krakowiak, 2019). Whereas contrastive upward state social comparison on social media has negative effects on psychological well-being, assimilative upward state social comparison on social media has positive effects on psychological well-being (e.g., Meier et al., 2020; Tosun & Kașdarma, 2020).

Despite this, the majority of research (especially research in the 2010s) just does not realize that there are actually two different subtypes of upward state social comparison on social media (for a review, see Meier & Johnson, 2022; Verduyn et al., 2017). This could explain why the majority of research (especially in the 2010s) nearly always finds only negative effects of upward state social comparison on psychological well-being. As a recent meta-analysis has shown, on social media, the more common subtype of upward state social comparison is contrastive upward state social comparison (McComb et al., 2023). Therefore, when researchers do not realize and specify the different subtypes of upward state social comparison, the more common subtype of upward state social comparison on social media is contrastive upward state social comparison, and thus, negative effects on psychological well-being are often observed.

More recently (in the late 2010s and early 2020s), some researchers started to notice the two different types of upward state social comparison, and started to call for the attention to differentiate between them (e.g., Latif et al., 2021; Meier & Schäfer, 2018; Meier et al., 2020). As these more recent studies have shown, besides the more common type of contrastive upward state social comparison and its negative effects, assimilative upward state social comparison does also happen on social media and could actually have positive effects on psychological well-being (e.g., Latif et al., 2021; Meier et al., 2020; Meier & Schäfer, 2018; Noon & Meier, 2019; Tosun & Kașdarma, 2020). Thus, both assimilative and contrastive upward state social comparison can happen on social media, resulting in very different effects on psychological well-being.

If researchers do not differentiate the nuances between assimilative and contrastive upward state social comparison, as mentioned above, they would probably only reveal the negative effects of upward state social comparison on psychological well-being, and fail to reveal the positive effects (for a review, see Meier & Johnson, 2022). As a result, our understandings on the relationships between upward state social comparison and psychological well-being would be only partial and incomplete. Thus, it is important for researchers to differentiate between the two different subtypes of upward state social comparison, so that researchers can more thoroughly unravel the relationships between upward state social comparison and psychological well-being.

#### 1.3.5. Emotional Consequences as Substitutions for Social Comparison

As mentioned above, some recent studies (e.g., Latif et al., 2021; Meier et al., 2020; Meier & Schäfer, 2018; Noon & Meier, 2019; Tosun & Kașdarma, 2020) have started to differentiate the nuances between assimilative and contrastive upward state social comparison. While these studies are indeed useful, they also have a measurement issue. That is, these studies do not measure the two subtypes of upward state social comparison themselves. Rather, these studies measure the emotional consequences of the two subtypes of upward state social comparison, and use the emotional consequences as substitutions for measuring the two subtypes of upward state social comparison.

For example, benign envy and malicious envy are, respectively, the emotional consequences of assimilative and contrastive upward state social comparison. Benign envy refers to one’s feeling that they could catch up with those superior others to have better accomplishments in the future (Wenninger et al., 2021). During assimilative upward state social comparison, individuals compare themselves with those superior others to figure out how they could be similar to them. After this type of comparison, individuals would already have ideas/plans of how they could catch up with those superior others, and thus could feel benign envy. On the other hand, malicious envy refers to one’s hostile feelings toward those superior others, wishing those superior others to lose their advantages (Noon & Meier, 2019). During contrastive upward state social comparison, individuals compare themselves with those superior others to see how they are different from them. After this type of comparison, individuals realize that there are indeed gaps between themselves and those superior others, and thus could feel malicious envy. Given these relationships between the two subtypes of upward state social comparison and their respective emotional consequences of envy, some researchers do not measure the two subtypes of upward state social comparison themselves, but rather measure the two types of envy as substitutions (e.g., see Latif et al., 2021; Meier et al., 2020; Meier & Schäfer, 2018; Noon & Meier, 2019).

Besides benign and malicious envy, other researchers have argued that after assimilative upward state social comparison, individuals would also have the feelings of optimism, admiration, and inspiration; after contrastive upward state social comparison, individuals would also have the feelings of resentment, depression, and shame (e.g., Smith, 2000; Tosun & Kașdarma, 2020). Thus, some researchers also measure those feelings as substitutions of measuring assimilative and contrastive upward state social comparison (e.g., Park & Baek, 2018; Tosun & Kașdarma, 2020).

However, it should be noted that emotional consequences occur after social comparison; they are not social comparisons per se (Johnson, 2021; Meier & Johnson, 2022). Therefore, the emotional consequences of social comparison should not be the substitutions of measuring social comparison. When researchers use emotional consequences as substitutions, the findings in existing research could be confounded. That is, the observed relationships between social media use and psychological well-being might not be clearly attributed to assimilative and contrastive upward state social comparison but rather attributed to their emotional consequences.

In summary, there are a total of five measurement issues of upward state social comparison, which could confound the findings in existing research (Johnson, 2021; Meier & Johnson, 2022). Therefore, it is critical to address and solve these measurement issues. In the next section, addressing all five measurement issues discussed above, we provide specific suggestions on how to measure upward state social comparison, and accordingly, we also develop specific measurement scales.

### 1.4. Measurement Recommendations and Proposed Measurement Scales

To address all aforementioned five measurement issues, we recommend researchers to consider the following five things together when measuring upward state social comparison on social media: (1) use ability-based items; (2) specify the upward directionality in the measurement items; (3) measure at the state level; (4) differentiate between assimilative and contrastive upward state social comparison; and (5) avoid using emotional consequences as substitutions.

Accordingly, based on these five suggestions, we develop a scale that clearly measures ability-based assimilative upward state social comparison and a scale that clearly measures ability-based contrastive upward state social comparison. In the next section, we will validate these two measurement scales, testing their reliability, and convergent, divergent, and predictive validity.

## 2. Validation Study

### 2.1. Reliability

In existing research, the scales of measuring upward state social comparison have demonstrated relatively high reliability with α ranging from about 0.70 to about 0.90 (e.g., Meier et al., 2020; Tosun & Kașdarma, 2020; Zheng et al., 2020). However, as reviewed previously, the measurement scales of upward state social comparison in existing research face multiple issues. Therefore, it remains uncertain what the reliability is of the scales that clearly measure upward state social comparison. In the current study, we propose a measurement scale of ability-based assimilative upward state social comparison and a measurement scale of ability-based contrastive upward state social comparison. Regarding the reliability of these two scales, we ask the following two research questions:

**RQ1a**: *What is the reliability of the ability-based assimilative upward state social comparison scale?*

**RQ1b**: *What is the reliability of the ability-based contrastive upward state social comparison scale?*

### 2.2. Convergent and Divergent Validity

Drawing from the relevant literature (e.g., Kang & Liu, 2019; McComb et al., 2023; Noon & Meier, 2019), we choose three variables to test the convergent and divergent validity of the two proposed scales—(1) trait ability-based social comparison, (2) age, and (3) perceived similarity with superior others.

First, trait ability-based social comparison should be positively related with both ability-based assimilative upward state social comparison and ability-based contrastive upward state social comparison. That is, if an individual has a general inclination to compare their ability with others (trait), they would also be likely to compare their ability with those superior others on social media to see how their ability could be similar to those superior others and how their ability is different from those superior others, when they see other people’s content on social media in the moment (state). Existing research has also suggested that people high in ability-based social comparison would be more likely to engage in ability-based assimilative and contrastive upward state social comparison on social media (e.g., Noon & Meier, 2019). Therefore, to demonstrate convergent validity of the two proposed scales, we expect both scales to be positively correlated with trait ability-based social comparison.

**H1a.**  *The ability-based assimilative upward state social comparison scale is positively corelated with trait ability-based social comparison.*

**H1b.**  *The ability-based contrastive upward state social comparison scale is positively corelated with trait ability-based social comparison.*

Second, results of recent meta-analysis research suggest that age is not related with the tendency of engaging in either ability-based assimilative or ability-based contrastive upward state social comparison on social media (McComb et al., 2023). Therefore, we use age as a factor to demonstrate divergent validity of the two proposed scales.

**H2a.**  *The ability-based assimilative upward state social comparison scale is not correlated with age.*

**H2b.**  *The ability-based contrastive upward state social comparison scale is not correlated with age.*

Lastly, if individuals compare themselves with those superior others to see how their ability could be similar to that of superior others, they are likely to actually think that they are indeed similar to them; on the other hand, if individuals compare themselves with those superior others to see how their ability is different from that of superior others, they are likely to actually think that they are indeed not similar to them (e.g., Kang & Liu, 2019; Noon & Meier, 2019). Therefore, perceived similarity with superior others should be positively related with the ability-based assimilative upward state social comparison scale (demonstrating convergent validity of this scale), and is not related with the ability-based contrastive upward state social comparison scale (demonstrating divergent validity of this scale).

**H3a.**  *The ability-based assimilative upward state social comparison scale is positively corelated with perceived similarity with superior others.*

**H3b.**  *The ability-based contrastive upward state social comparison scale is not correlated with perceived similarity with superior others.*

### 2.3. Predictive Validity

As reviewed above, engaging in ability-based assimilative and contrastive upward state social comparison would, respectively, have positive and negative effects on psychological well-being (e.g., Meier et al., 2020). Therefore, to examine the predictive validity of the two scales, we focus on how these two scales predict psychological well-being outcomes. Existing research has generally focused on three psychological well-being outcomes—(1) positive/negative affect, (2) benign/malicious envy, and (3) life satisfaction (e.g., Meier et al., 2020; Meier & Schäfer, 2018; Tosun & Kașdarma, 2020). Based on what existing studies have suggested, we hypothesize that the ability-based assimilative upward state social comparison scale positively predicts all three psychological well-being outcomes (i.e., more positive than negative affect, more benign than malicious envy, higher life satisfaction), whereas the ability-based contrastive upward state social comparison scale negatively predicts all three psychological well-being outcomes (i.e., more negative than positive affect, more malicious than benign envy, and lower life satisfaction). Specifically,

**H4a.**  *The ability-based assimilative upward state social comparison scale predicts more positive affect than negative affect.*

**H4b.**  *The ability-based contrastive upward state social comparison scale predicts more negative affect than positive affect.*

**H5a.**  *The ability-based assimilative upward state social comparison scale predicts more benign envy than malicious envy.*

**H5b.**  *The ability-based contrastive upward state social comparison scale predicts more malicious envy than benign envy.*

**H6a.**  *The ability-based assimilative upward state social comparison scale predicts higher life satisfaction.*

**H6b.**  *The ability-based contrastive upward state social comparison scale predicts lower life satisfaction.*

## 3. Materials and Methods

### 3.1. Participants

We recruited a total of 479 undergraduate students from a large university on the west coast of the U.S. to participate in the study. A total of 17 participants were excluded from data analyses, because they were not users of any social media platforms (10 participants), they spent an unusually long time completing the study (i.e., five participants spent two standard deviations above the average time completing the study), or they potentially did not pay enough attention (two participants). The final sample size was 462 participants. Most participants were female (*n* = 349, 75.54%), with others being male (*n* = 105, 22.73%) or other genders (*n* = 4, 0.87%), and with four participants (0.87%) preferring not to reveal their gender. Participants identified themselves as Asian Americans (*n* = 155, 33.55%), or Caucasian/White (*n* = 87, 18.83%), or Hispanic/Latino (*n* = 81, 17.53%), or other race/ethnicity (*n* = 76, 16.45%), or multi-racial (*n* = 48, 10.39%), or African American (*n* = 9, 1.95%), or Pacific Islander (*n* = 5, 1.08%), or Native American (*n* = 1, 0.22%). Participants’ ages ranged from 18 to 42 years old (*M* = 19.75, *SD* = 2.09).

### 3.2. Procedure

The study was approved by the Institutional Review Board of University of California, Davis. Informed consent was obtained for all participants. After agreeing to participate in the study, participants were directed to an online Qualtrics survey. Participants started with answering questions that measured their trait ability-based social comparison. Then, participants were asked to recall their most recent social media use (i.e., the last time they were using social media). While recalling the last time they used social media, participants were asked to report their ability-based assimilative and contrastive upward state social comparison, perceived similarity with superior others, and also, their psychological well-being. Lastly, participants answered demographic questions.

### 3.3. Measures

#### 3.3.1. Trait Ability-Based Social Comparison

Participants answered six items in the Iowa-Netherlands Comparison Orientation Measure (INCOM) scale (Gibbons & Buunk, 1999) to report their trait ability-based social comparison. Participants answered the six items on a 5-point Likert scale (1 = strongly disagree, 5 = strongly agree). A sample item was “in general, I often compare myself with others with respect to what I have accomplished in life”. After adjusting for the reverse-coded item, a 6-item scale was calculated by averaging the six questions, with higher scores indicating higher trait ability-based social comparison (*α* = 0.82, *M* = 3.76, *SD* = 0.76).

#### 3.3.2. Ability-Based Assimilative and Contrastive Upward State Social Comparison

Based on the five specific measurement suggestions we recommended in the previous sections, we developed a 6-item scale of ability-based assimilative upward state social comparison, and a 6-item scale of ability-based contrastive upward state social comparison. In the following paragraphs, we will describe, step by step, how we developed the two measurement scales.

To develop the scale of ability-based assimilative upward state social comparison, first, based on our recommendation that ability-based social comparison items should be used, we chose the six ability-based social comparison measurement items in the Iowa-Netherlands Comparison Orientation Measure (INCOM) scale (Gibbons & Buunk, 1999). Those six items (on a 5-point Likert scale: 1 = strongly disagree, 5 = strongly agree) are widely used in existing research to measure ability-based social comparison and have been empirically tested to be highly valid and reliable (Gibbons & Buunk, 1999). Second, based on our recommendation that the upward directionality needs to be specified, in each of the six items, we added the phrase “compare… with others who are superior to me”, so that each item reflected the upward directionality of social comparison (e.g., Gerber et al., 2018). Third, based on our recommendation that the assimilative subtype of upward social comparison should be specified, in each of the six items, we added the phrase “…to focus on how I can be similar to the superior others”, so that each item reflected assimilative upward social comparison (e.g., Tsay-Vogel & Krakowiak, 2019). Fourth, based on our recommendation that emotional consequences should not be the substitutions for measurement, for each of the six items, we checked to ensure that no emotions (e.g., feelings of benign envy, inspiration) were included. Lastly, based on our recommendation that the state level should be measured, we carried out the following two things—first, we ensured that in each of the six items, there was no “trait-like” expressions, such as “always, general, often”, and second, in the measurement scale, we asked participants to answer each of the six items while recalling what happened at the moment when they saw other people’s social media content during the last time they were using social media. In other words, participants reported the extent to which they engaged in ability-based assimilative upward social comparison at the moment when they saw other people’s social media content during the last time they were using social media. Doing so can ensure that the state level was measured (see Choi and Kim (2021), Wirtz et al. (2021), Yu (2023) for a similar approach). After all these five steps (each step following one of our measurement recommendations), we finally developed a 6-item scale for measuring ability-based assimilative upward state social comparison (see Appendix A).

To develop the ability-based contrastive upward state social comparison scale, four of the five steps mentioned in the last paragraph were identical—we chose the six ability-based items, specified the upward directionality, made sure no emotional consequences were included, and made sure to measure at the state level. The only different step was that, this time, we needed to specify the contrastive subtype of upward social comparison. Therefore, in each of the six items, we added the phrase “…to focus on how I am different from the superior others”, so that each item reflected contrastive upward social comparison (e.g., Collins, 1996). After all these five steps, we finally developed a 6-item scale for measuring ability-based contrastive upward state social comparison. Participants reported the extent to which they engaged in ability-based contrastive upward social comparison at the moment when they saw other people’s social media content during the last time they were using social media (see Appendix A).

#### 3.3.3. Perceived Similarity with Superior Others

We measured perceived similarity with superior others using four items adopted from the Perceived Homophily Scale (McCroskey et al., 1975). On a 7-point Likert scale (1 = strongly disagree, 7 = strongly agree), participants reported that, during the last time they were using social media, right after they compared themselves with people who were superior to them, the extent to which they perceived that they were similar to those superior others (e.g., “people who were superior to me on social media were similar to me”). A 4-item scale was formed by averaging the four items (*α* = 0.84, *M* = 3.41, *SD* = 1.17). Higher scores demonstrated a higher perceived similarity with superior others.

#### 3.3.4. Positive and Negative Affect

To measure positive and negative affect, we adopted six questions from existing research that measure individual’s positive and negative feelings after they engage in ability-based assimilative and contrastive upward state social comparison (e.g., Park & Baek, 2018; Smith, 2000; Tosun & Kașdarma, 2020). Specifically, on a 5-point Likert scale (1 = very slightly or not at all, 5 = extremely), participants indicated, during the last time they were using social media, right after comparing themselves with people who were superior to them, in the moment, the degree that they had three positive affects (1. inspiration, 2. optimism, 3. admiration), and three negative affects (4. shame, 5. depression, and 6. resentment). We calculated a scale for positive affect by averaging the scores of inspiration, optimism, and admiration (*α* = 0.78, *M* = 2.61, *SD* = 1.03), and a scale for negative affect by averaging the scores of shame, depression, and resentment (*α* = 0.76, *M* = 1.90, *SD* = 0.96).

To capture which type of affect individuals had most (relative to the other type), we created an “affect differences scale” by subtracting the negative affect scale from the positive affect scale, such that positive scores demonstrated more positive (than negative) affect, and negative scores demonstrated more negative (than positive) affect (*M* = 0.71, *SD* = 1.36, *Min* = −3.67, *Max* = 4.00).

#### 3.3.5. Benign and Malicious Envy

In the present study, we used the scale by Lange and Crusius (2015) to measure benign and malicious envy. On a 6-point Likert scale (1 = strongly disagree, 6 = strongly agree), participants indicated, during the last time they were using social media, right after comparing themselves with people who were superior to them, in the moment, the degree that they felt benign envy (four items, e.g., “I noticed that those superior people were better than me, and I tried to improve myself), and malicious envy (five items, e.g., “I envied those superior people and I felt ill will toward them). We created a 4-item scale for benign envy by calculating the mean of its four items (*α* = 0.82, *M* = 3.59, *SD* = 1.13), and we created a 5-item scale for malicious envy by calculating the mean of its five items (*α* = 0.92, *M* = 2.25, *SD* = 1.12).

To capture which type of envy individuals had most (relative to the other type), we calculated an “envy differences scale” by subtracting the malicious envy scale from the benign envy scale, such that positive scores indicated more benign (than malicious) envy, and negative scores indicated more malicious (than benign) envy (*M* = 1.34, *SD* = 1.32, *Min* = −1.85, *Max* = 5.00).

#### 3.3.6. Life Satisfaction

To measure life satisfaction, we used the Satisfaction with Life Scale by Diener et al. (1985). Participants reported, during the last time they were using social media, right after comparing themselves with people who were superior to them, in the moment, the extent to which they were satisfied with their life. Participants answered four items on a 7-point Likert scale (1 = strongly disagree, 7 = strongly agree) (e.g., “I was satisfied with my life”). We created a 4-item scale by calculating the mean of the four questions. Higher scores demonstrated higher life satisfaction (*α* = 0.83, *M* = 4.02, *SD* = 1.37).

## 4. Results

### 4.1. Confirmatory Factor Analysis (CFA)

To test the model fit of the two proposed scales, after adjusting for reverse-coded items, we conducted a confirmatory factor analysis (CFA) on the six items of the ability-based assimilative upward state social comparison scale and the six items of the ability-based contrastive upward state social comparison scale. The model fit was not acceptable. After respectively dropping two items from each scale, the model fit was acceptable—CMIN/DF = 4.13, GFI = 0.96, TLI = 0.95, CFI = 0.97, RMSEA = 0.08, 90% CI [0.06, 0.10]. As a result, we created a 4-item scale for ability-based assimilative upward state social comparison by averaging its four items (*α* = 0.86, *M* = 3.14, *SD* = 0.94). Higher scores demonstrated higher engagement in ability-based assimilative upward state social comparison. Likewise, we created a 4-item scale for ability-based contrastive upward state social comparison by averaging its four items (*α* = 0.85, *M* = 3.06, *SD* = 0.93), with higher scores indicating higher engagement in ability-based contrastive upward state social comparison. These two 4-item scales were used for all subsequent analyses on reliability, and convergent, divergent, and predictive validity.

### 4.2. Reliability

To examine the reliability of the two proposed scales (RQ1a and RQ1b), we used SPSS (version 29) to calculate the Cronbach’s alphas. Results showed that the reliability of the ability-based assimilative upward state social comparison scale was *α* = 0.86, and the reliability of the ability-based contrastive upward state social comparison scale was *α* = 0.85. Thus, both scales demonstrated relatively high reliability.

### 4.3. Convergent and Divergent Validity

To test convergent and divergent validity (i.e., H1–H3), we calculated bivariate correlations between the ability-based assimilative upward state social comparison scale, the ability-based contrastive upward state social comparison scale, trait ability-based social comparison, age, and perceived similarity with superior others (see Table 1). Results showed that trait ability-based social comparison was both positively correlated with the ability-based assimilative upward state social comparison scale (*r* = 0.28, *p* < 0.001) and positively correlated with the ability-based contrastive upward state social comparison scale (*r* = 0.28, *p* < 0.001), Therefore, H1a and H1b were supported, demonstrating the convergent validity of both scales.

In terms of H2a and H2b, results showed that age was neither correlated with the ability-based assimilative upward state social comparison scale (*r* = 0.01, *p* = 0.866), nor correlated with the ability-based contrastive upward state social comparison scale (*r* = −0.01, *p* = 0.883). Thus, H2a and H2b were supported, demonstrating the divergent validity of the two scales.

For the relationships between perceived similarity with superior others and the two proposed scales (H3a and H3b), results showed that perceived similarity with superior others was positively related with the ability-based assimilative upward state social comparison scale (*r* = 0.25, *p* < 0.001), supporting H3a and demonstrating the convergent validity of this scale. On the other hand, perceived similarity with superior others was not related with the ability-based contrastive upward state social comparison scale (*r* = 0.04, *p* = 0.452), supporting H3b and demonstrating the divergent validity of this scale.

### 4.4. Predictive Validity

To examine how the two proposed scales predicted three components of psychological well-being (i.e., positive/negative affect (H4), benign/malicious envy (H5), life satisfaction (H6)), we ran three linear regressions. The first regression examined how the two proposed scales predicted positive/negative affect (H4). In this regression, the predictors were the two proposed scales, and the dependent variable was affect differences (i.e., positive affect minus negative affect). Results showed that the ability-based assimilative upward state social comparison scale positively predicted affect differences, B = 0.43, *t* (459) = 5.68, *p* < 0.001, meaning that the ability-based assimilative upward state social comparison scale predicted more positive than negative affect, supporting H4a; on the other hand, the ability-based contrastive upward state social comparison scale negatively predicted affect differences (B = −0.50, *t* (459) = −6.45, *p* < 0.001), meaning that the ability-based contrastive upward state social comparison scale predicted more negative than positive affect, supporting H4b.

In the second regression, we examined how the two proposed scales predicted benign/malicious envy (H5). In this regression, the predictors were still the two proposed scales, and the dependent variable this time was envy differences (i.e., benign envy minus malicious envy). Results showed that the ability-based assimilative upward state social comparison scale positively predicted envy differences (B = 0.54, *t* (459) = 7.38, *p* < 0.001), supporting H5a––the ability-based assimilative upward state social comparison scale predicted more benign than malicious envy. On the other hand, the ability-based contrastive upward state social comparison scale negatively predicted envy differences (B = −0.17, *t* (459) = −2.23, *p* = 0.026), supporting H5b––the ability-based contrastive upward state social comparison scale predicted more malicious than benign envy.

The third regression examined how the two proposed scales predicted life satisfaction (H6). The predictors in this regression were still the two proposed scales, and the dependent variable this time was life satisfaction. Results demonstrated that the ability-based assimilative upward state social comparison scale predicted higher life satisfaction (B = 0.22, *t* (459) = 2.76, *p* = 0.006), supporting H6a; the ability-based contrastive upward state social comparison scale predicted lower life satisfaction (B = −0.34, *t* (459) = −4.19, *p* < 0.001), supporting H6b.

Overall, the results of regression analyses demonstrated that the ability-based assimilative upward state social comparison scale positively predicted all three components of psychological well-being, whereas the ability-based contrastive upward state social comparison scale negatively predicted all three components of psychological well-being. Therefore, these results indeed demonstrated the predictive validity of the two proposed scales.

## 5. Discussion

In this paper, addressing multiple measurement issues of upward state social comparison in the literature of social media use and psychological well-being, we provided multiple specific suggestions on how to measure upward state social comparison. We also developed two measurement scales, and the results of the validation study showed that a 4-item scale of ability-based assimilative upward state social comparison and a 4-item scale of ability-based contrastive upward state social comparison demonstrated good reliability, and convergent, divergent, and predictive validity.

### 5.1. Measurement Issues and Recommendations

As reviewed in the previous sections, in the existing literature of social media use and psychological well-being, there are multiple issues involving the measurement of upward state social comparison, including that it (1) conceptualizes upward state social comparison as ability-based but measures it using both ability-based and opinion-based items, (2) does not specify the upward directionality when measuring upward state social comparison, (3) does not measure upward state social comparison as a state but as a trait, (4) fails to differentiate the two different subtypes of upward state social comparison (assimilative and contrastive), and (5) uses the emotional consequences of upward state social comparison as substitutions for measuring upward state social comparison.

To address these five measurement issues, we provided five specific measurement suggestions. Specifically, in measuring upward state social comparison, we recommended researchers to (1) use ability-based items; (2) specify the upward directionality in the measurement items; (3) specify that upward state social comparison happens at the state level; (4) specify the differences between assimilative and contrastive upward state social comparison; and (5) make sure emotional consequences are not substitutions of measuring upward state social comparison. Based on these five recommendations, we proposed a 6-item scale of ability-based assimilative upward state social comparison and a 6-item scale of ability-based contrastive upward state social comparison.

### 5.2. Validation of the Proposed Measurement Scales

In our validation study, based on the results of the confirmatory factor analysis (CFA), we dropped two items from each of the two proposed scales. As a result, the 4-item ability-based assimilative upward state social comparison scale and the 4-item ability-based contrastive upward state social comparison scale demonstrated good model fit in CFA. We first tested the reliability of the two 4-item scales, and results showed that both scales demonstrated relatively high reliability (Cronbach’s alphas around 0.85).

Furthermore, the two 4-item scales demonstrated convergent and divergent validity. Specifically, consistent with what existing research has suggested (e.g., Noon & Meier, 2019), individuals high in trait ability-based social comparison indeed were also more likely to engage in both ability-based assimilative and contrastive upward state social comparison, indicating the convergent validity of both scales. Moreover, consistent with what existing research has suggested (e.g., McComb et al., 2023), age was not related with either ability-based assimilative or contrastive upward state social comparison, demonstrating the divergent validity of both scales. Lastly, perceived similarity with superior others was positively related with ability-based assimilative upward state social comparison (demonstrating convergent validity of this scale), and perceived similarity with superior others was not related with ability-based contrastive upward state social comparison (demonstrating divergent validity of this scale). Overall, these results showed that the two 4-item scales indeed demonstrated both convergent and divergent validity.

Lastly, to test the predictive validity of the two 4-item scales, we examined the relationships between the two scales and three psychological well-being outcomes (i.e., (1) positive/negative affect, (2) benign/malicious envy, and (3) life satisfaction). Consistent with what existing research has suggested (e.g., Meier et al., 2020; Tosun & Kașdarma, 2020), the ability-based assimilative upward state social comparison scale indeed positively predicted all three components of psychological well-being (i.e., more positive than negative affect, more benign than malicious envy, and higher life satisfaction), and the ability-based contrastive upward state social comparison scale indeed negatively predicted all three components of psychological well-being (i.e., more negative than positive affect, more malicious than benign envy, and lower life satisfaction). These results indicated that these two scales indeed had good predictive validity.

In summary, results of the validation study showed that the 4-item scale of ability-based assimilative upward state social comparison and the 4-item scale of ability-based contrastive upward state social comparison we developed were reliable and also demonstrated various forms of validity (divergent, convergent, and predictive validity).

### 5.3. Strengths, Limitations, and Future Research Directions 

A limitation of this paper is that we only validated the proposed scales in a sample that consisted of mostly young adult females in the U.S. (notably, 75% of our sample is female). It is unclear whether and how the two proposed scales would work in other gender, age, and cultural groups. Thus, we encourage future research to further validate the proposed scales in a more diverse sample and in other populations. Another limitation of the current study is that, in the scales we developed, we asked participants to report their ability-based assimilative and contrastive upward state social comparison while recalling what happened at the moment when they saw other people’s content during the last time they were using social media. This method (i.e., asking participants to report their social comparison behaviors while recalling what happened during/since the last time they were using social media) has been implemented in existing research to measure social comparison on social media at the state level (e.g., see Choi & Kim, 2021; Wirtz et al., 2021; Yu, 2023). However, it should be acknowledged that participants might not accurately recall what exactly happened during the last time they were using social media, and therefore, participants’ responses might not be very accurate. Future research could validate the proposed scales in a context where participants are actually using social media in the moment, rather than asking participants to recall what happened during the last time they were using social media.

A limitation of this paper is that we only validated the proposed scales in a sample that consisted of mostly young adult females in the U.S. (notably, 75% of our sample is female). It is unclear whether and how the two proposed scales would work in other gender, age, and cultural groups. Thus, we encourage future research to further validate the proposed scales in a more diverse sample and in other populations. Another limitation of the current study is that, in the scales we developed, we asked participants to report their ability-based assimilative and contrastive upward state social comparison while recalling what happened at the moment when they saw other people’s content during the last time they were using social media. This method (i.e., asking participants to report their social comparison behaviors while recalling what happened during/since the last time they were using social media) has been implemented in existing research to measure social comparison on social media at the state level (e.g., see Choi & Kim, 2021; Wirtz et al., 2021; Yu, 2023). However, it should be acknowledged that participants might not accurately recall what exactly happened during the last time they were using social media, and therefore, participants’ responses might not be very accurate. Future research could validate the proposed scales in a context where participants are actually using social media in the moment, rather than asking participants to recall what happened during the last time they were using social media.

Despite these limitations, this paper still makes important contributions to the literature. As we noted previously, due to multiple measurement issues of upward state social comparison, the research that uses flawed measurements could provide potentially inaccurate results about the relationships between social media use, upward state social comparison, and psychological well-being (Johnson, 2021; Meier & Johnson, 2022). In this paper, addressing multiple measurement issues together, we proposed measurement scales of ability-based assimilative and contrastive upward state social comparison. In our validation study, we showed that the two 4-item scales we proposed were valid and reliable, providing solutions to these measurement issues. Additionally, in our validation study, with valid and reliable measurements, we showed that ability-based assimilative and contrastive upward state social comparison on social media are indeed, respectively, related with positive and negative psychological well-being. Therefore, with valid and reliable measurements, our paper provides more accurate and clearer evidence to support that ability-based assimilative and contrastive upward state social comparison are indeed important variables that could explain the relationships between social media use and psychological well-being.

## 6. Conclusions

In conclusion, the current paper provides useful measurement suggestions and tools, disentangling the measurement issues of upward state social comparison on social media. We hope that future research could more clearly and accurately unravel the relationships between social media use, upward state social comparison, and psychological well-being, ultimately helping social media users improve their psychological well-being.

## Figures and Tables

**Table 1:** Bivariate correlation analyses results in the validation study.

	1	2	3	4	5
1. Assimilative upward state					
2. Contrastive upward state	0.54 ***				
3. Trait ability-based	0.28 ***	0.28 ***			
4. Age	0.01	−0.01	0.05		
5. Perceived similarity	0.25 ***	0.04	−0.01	−0.06	

Note. *** *p* < 0.001. Assimilative upward state = ability-based assimilative upward state social comparison. Contrastive upward state = ability-based contrastive upward state social comparison. Trait ability-based = trait ability-based social comparison. Perceived similarity = perceived similarity with superior others.

## Data Availability

The data presented in this study are available on request from the corresponding author due to privacy restrictions.

## Appendix A

Proposed Measurement Scales of Ability-Based Assimilative Upward State Social Comparison and Ability-Based Contrastive Upward State Social Comparison. Instructions: the following questions ask you to indicate how you compared yourself with people whom you perceived to be superior to you, during your **most recent** social media use. Recall **the last time when you were using social media**, which could be 5 min ago, 30 min ago, an hour ago, several hours ago, or yesterday…, and answer the following questions on a 5-point scale (1 = Strongly Disagree, 5 = Strongly Agree) **The last time I used social media, when I saw the content posted by others, at the moment,** 1. I compared how I was doing with how others who were superior to me were doing to: (a) focus on how I could be similar to the superior others. (b) focus on how I was different from the superior others. 2. I paid a lot of attention to how I did things compared with how others who were superior to me did things to: (a) focus on how I could be similar to the superior others. (b) focus on how I was different from the superior others. 3. I compared what I had done with how others who were superior to me had done: (a) in order to find out how I could be similar to the superior others. (b) in order to find out how I was different from the superior others. 4. I compared how I was doing socially (e.g., social skills, popularity) with others who were superior to me to: (a) focus on how my social skills and popularity could be similar to the superior others. (b) focus on how my social skills and popularity were different from the superior others. 5. (a) *I **did not** compare with others who were superior to me to focus on how I could be similar to the superior others. (b) *I **did not** compare with others who were superior to me to focus on how I was different from the superior others. 6. I compared myself with others who were superior to me to: (a) focus on how I could attain the similar life accomplishments of the superior others. (b) focus on how my life accomplishments were different from the life accomplishments of the superior others. *Note*. *Item 5a and 5b are reverse-coded items. The proposed six measurement items of ability-based assimilative upward state social comparison were—1a, 2a, 3a, 4a, 5a, 6a. The proposed six measurement items of ability-based contrastive upward state social comparison were—1b, 2b, 3b, 4b, 5b, 6b. In the validation study, the four items of ability-based assimilative upward state social comparison were 1a, 2a, 3a, 6a, and the four items of ability-based contrastive upward state social comparison were 1b, 2b, 3b, 6b.
